# Autonomic Nervous System Dysfunction Is Associated With Re-hospitalization in Pediatric Septic Shock Survivors

**DOI:** 10.3389/fped.2021.745844

**Published:** 2022-01-04

**Authors:** Colleen M. Badke, Lindsey Swigart, Michael S. Carroll, Debra E. Weese-Mayer, L. Nelson Sanchez-Pinto

**Affiliations:** ^1^Division of Critical Care Medicine, Ann & Robert H. Lurie Children's Hospital of Chicago, Chicago, IL, United States; ^2^Department of Pediatrics, Northwestern University Feinberg School of Medicine, Chicago, IL, United States; ^3^Stanley Manne Children's Research Institute, Chicago, IL, United States; ^4^Data Analytics and Reporting, Ann & Robert H. Lurie Children's Hospital of Chicago, Chicago, IL, United States; ^5^Division of Autonomic Medicine, Ann & Robert H. Lurie Children's Hospital of Chicago, Chicago, IL, United States

**Keywords:** critical care, pediatrics, sepsis, autonomic nervous system, outcomes

## Abstract

**Objective:** Re-hospitalization after sepsis can lead to impaired quality of life. Predictors of re-hospitalization could help identify sepsis survivors who may benefit from targeted interventions. Our goal was to determine whether low heart rate variability (HRV), a measure of autonomic nervous system dysfunction, is associated with re-hospitalization in pediatric septic shock survivors.

**Materials and Methods:** This was a retrospective, observational cohort study of patients admitted between 6/2012 and 10/2020 at a single institution. Patients admitted to the pediatric intensive care unit with septic shock who had continuous heart rate data available from the bedside monitors and survived their hospitalization were included. HRV was measured using age-normalized z-scores of the integer HRV (HRVi), which is the standard deviation of the heart rate sampled every 1 s over 5 consecutive minutes. The 24-h median HRVi was assessed on two different days: the last 24 h of PICU admission (“last HRVi”) and the 24-h period with the lowest median HRVi (“lowest HRVi”). The change between the lowest and last HRVi was termed “delta HRVi.” The primary outcome was re-hospitalization within 1 year of discharge, including both emergency department encounters and hospital readmission, with sensitivity analyses at 30 and 90 days. Kruskal-Wallis, logistic regression, and Poisson regression evaluated the association between HRVi and re-hospitalizations and adjusted for potential confounders.

**Results:** Of the 463 patients who met inclusion criteria, 306 (66%) were re-hospitalized, including 270 readmissions (58%). The last HRVi was significantly lower among re-hospitalized patients compared to those who were not (*p* = 0.02). There was no difference in the lowest HRVi, but patients who were re-hospitalized showed a smaller recovery in their delta HRVi compared to those who were not re-hospitalized (*p* = 0.02). This association remained significant after adjusting for potential confounders. In the sensitivity analysis, a smaller recovery in delta HRVi was consistently associated with a higher likelihood of re-hospitalization.

**Conclusion:** In pediatric septic shock survivors, a smaller recovery in HRV during the index admission is significantly associated with re-hospitalization. This continuous physiologic measure could potentially be used as a predictor of patients at risk for re-hospitalization and lower health-related quality of life.

## Introduction

Sepsis is a leading cause of morbidity and mortality worldwide (1–3). Up to 35% of pediatric sepsis survivors experience deterioration in their health-related quality of life (HRQL) for at least 1 year following hospitalization (4–6). Children who experience severe sepsis and septic shock are also more likely to utilize the healthcare system subsequently (7, 8). Certain factors associated with a sepsis admission increase the risk of death or deterioration in HRQL at 3 months, including renal replacement therapy, extracorporeal life support, and cardiopulmonary resuscitation (9). Certain biomarkers (specifically, the PERSEVERE tool which includes C-C chemokine ligand 3 (CCL3), interleukin 8 (IL8), heat shock protein 70 kDa 1B, granzyme B, and matrix metallopeptidase 8) have also been shown to predict HRQL at 3 months (10). While measures of autonomic nervous system (ANS) function have been used to risk-stratify critically ill patients at risk of poor outcomes (11, 12), it is unknown if these measures are associated with risk of readmission or lower HRQL in survivors.

The ANS is a unique system as it regulates functions in all organ systems, maintaining homeostasis when confronted with stressors, including sepsis (13). ANS dysfunction (ANSD) may occur during an imbalanced or maladaptive response to infection, and is often identified as excessive, uncontrolled, or prolonged sympathetic activation, or inappropriate regulation by the parasympathetic nervous system (14). Most of the evidence of ANSD in critical illness is based on measures of heart rate variability (HRV), a non-invasive, continuous physiologic marker of ANS function that is inversely correlated with organ dysfunction and mortality in both adult and pediatric patients (11, 15–17). The goal of this study was to assess whether HRV during an index admission for septic shock was associated with re-hospitalizations within a year in survivors.

## Materials and Methods

### Study Design and Patient Population

This was a retrospective, observational cohort study from 6/2012 to 10/2020 at a single institution. Patients admitted to the pediatric intensive care unit (PICU) with septic shock who had heart rate data available from bedside monitors were included in the analysis. Septic shock was defined as the need for vasoactive medications in children with confirmed or suspected infection within 72 h of PICU admission. The study was approved with a waiver of consent by Ann & Robert H. Lurie Children's Hospital of Chicago institutional review board (IRB #2020-3868).

### HRV Measurement

Continuous heart rate data from the entire PICU stay were extracted from bedside monitors using the BedMaster system (Hillrom, Jupiter, FL) and stored locally in an archiving system within hospital servers. HRV was estimated using the integer HRV (HRVi), which was calculated as the standard deviation of the heart rate sampled every 1 s over 5 consecutive minutes and normalized for age as previously described (11). A minimum of 50% of the heart rate samples in each 5-min interval were needed to be included in the analysis. Within each 5-min interval included, data availability was high, with >90% of intervals including >90% of possible heart rate samples. The median HRVi in 24 h was assessed on two different days: the last 24 h of PICU admission (“last HRVi,” a proxy for the pre-discharge level of ANSD) and the 24-h period with the lowest median HRVi during the PICU admission (“lowest HRVi,” a proxy for the most severe ANSD). The change between the lowest and the last HRVi was also calculated and termed “delta HRVi.”

### Outcomes

The primary outcome was all cause re-hospitalization to the same institution within 1 year from the index admission, including emergency department visits and hospital readmissions (both inpatient and PICU). The secondary outcome was the total number of re-hospitalizations (both inpatient and ICUs) within 1 year from the index admission to the same institution.

### Confounder Analysis

To address possible confounders of re-hospitalization, we used a multivariable regression analysis to adjust for age, severity of illness on admission using the Pediatric Risk of Mortality (PRISM) III (18), immunocompromised state, and chronic comorbid conditions. Immunocompromised status (defined as an oncologic diagnosis and/or transplantation) and chronic comorbid conditions were classified according to Feudtner's criteria (19). We defined multi-comorbidity as having 2 or more chronic comorbid conditions. We chose these confounders *a priori* based on common risk factors associated with poor outcomes in children with septic shock that may also influence HRV.

### Sensitivity Analysis

To determine if there were differences in the associations based on the timeframe of re-hospitalization, we performed a sensitivity analysis of patients re-hospitalized within 30 days and within 90 days from the index admission.

### Statistical Analysis

Data were analyzed using R version 3.6.1 (R Foundation for Statistical Computing, Vienna, Austria) (20). Kruskal-Wallis and chi-square tests, logistic regression (primary outcome), and Poisson regression (secondary outcome) were performed to evaluate the association between HRVi measurements and re-hospitalization.

## Results

There were 463 patients who met inclusion criteria during the study period. Of those patients, 306 (66%) were re-hospitalized within 1 year, and 270 (58%) were readmitted to an inpatient ward or an ICU. The demographics and clinical characteristics of these patients during their index admission are presented in Table 1. Of all the patients in this cohort, 34% were immunocompromised and 87% had multi-comorbidity. Patients who were re-hospitalized were more likely to be non-white and Hispanic/Latino and more likely to be immunocompromised (42.2 vs. 17.8%). There were no differences in age, sex, PRISM III, technology dependence, length of stay, inotrope-free days, or ventilator-free days at 28 days. Patients who were re-hospitalized had on average 3 re-hospitalizations (IQR 1–6) within the first year after the index admission. Respiratory and infection/sepsis were the main causes of re-hospitalization. Eighty-five (28%) patients were re-hospitalized within 30 days of the index admission, and 207 (68%) were re-hospitalized within 90 days of the index admission (Supplementary Table 1).

**Table 1:** Demographics of study population.

**Characteristics**	**No Re-hospitalization**	**Re-hospitalization**	***P*** -value^*^
	**(*n* = 157)**	**(*n* = 306)**	
Age (years), median (IQR)	9.2 (3.3–14.8)	7.9 (2.3–14.5)	0.26
Sex (male), *n* (%)	78 (49.7%)	171 (56%)	0.24
Race, *n* (%)			0.02
White	82 (52.5%)	117 (38.2%)	
Black/African American	24 (15.3%)	49 (16.0%)	
Asian	10 (6.4%)	22 (7.2%)	
Other/Unknovwn	41 (26.1%)	118 (38.6%)	
Ethnicity, *n* (%)			0.01
Not Hispanic/Latino	112 (71.3%)	181 (59.2%)	
Hispanic/Latino	41 (26.1%)	123 (40.2%)	
Declined/Unknown	4 (2.5%)	2 (0.7%)	
Length of stay (days), median (IQR)	11.9 (7.1–26.1)	15.8 (8.0–30.7)	0.07
PRISM III Score, median (IQR)	13.0 (6.0–19.0)	13.5 (8.0–20.0)	0.17
Immunocompromised state, *n* (%)	28 (17.8%)	129 (42.2%)	<0.001
**Number of chronic comorbid conditions**, ***n*** **(%)**			
0	9 (5.7%)	4 (1.3%)	0.02
1	23 (14.6%)	26 (8.5%)	0.06
2 or more	125 (79.6%)	276 (90.2%)	0.003
Ventilated, *n* (%)	103 (65.6%)	175 (57.2%)	0.10
Ventilator-free days, median (IQR)	24 (17–28)	25 (15–28)	0.57
Inotrope-free days, median (IQR)	26 (24–27)	26 (23–27)	0.89

* *Chi-square test performed for categorical variables. Kruskall-Wallis test performed for continuous variables. P-value < 0.05 considered significant*.

### Primary Outcome

The median last HRVi measurement during the index admission was significantly lower among patients who were re-hospitalized compared to those who were not (*p* = 0.02, Table 2). The median lowest HRVi measurement during the index admission was not significantly different between patients who were re-hospitalized compared to those who were not (*p* = 0.67), but patients who were re-hospitalized showed a smaller improvement in their delta HRVi (*p* = 0.02, Table 2).

**Table 2:** HRVi measurements for septic shock survivors.

	**Not Re-**	**Re-**	***P*** -value^*^
	**hospitalized**	**hospitalized**	
Last HRVi, median (IQR)	−0.216 (−0.525, 0.061)	−0.349 (−0.626, 0.009)	0.016
Lowest HRVi, median (IQR)	−0.931 (−1.269, −0.574)	−0.939 (−1.213, −0.544)	0.667
Delta HRVi, median (IQR)	0.579 (0.219, 1.030)	0.444 (0.135, 0.844)	0.024

* *Kruskal-Wallis test. P < 0.05 considered significant*.

After controlling for potential confounders (including age, PRISM III, immunocompromised state, and multi-comorbidity) using logistic regression, the median last and delta HRVi measurements remained significantly associated with re-hospitalization, with higher HRVi measurements associated with a lower adjusted odds ratio of re-hospitalization. The lowest HRVi measurement was not associated with re-hospitalization in the unadjusted or adjusted models (Table 3).

**Table 3:** Unadjusted and adjusted odds ratios (OR) for re-hospitalizations within 1 year after septic shock admission based on age-normalized HRVi.

**Predictor**	**Unadjusted**	**Adjusted**
	**OR (95% CI)**	**OR (95% CI)**
Last HRVi Measurement	0.62 (0.41–0.93)	0.57 (0.36–0.87)
Age		1.00 (1.00–1.00)
PRISM III		1.00 (0.98–1.03)
Immunocompromised state		3.46 (2.17–5.68)
Multi-comorbidity		2.24 (1.25–4.03)
Lowest HRVi Measurement	1.10 (0.69–1.74)	1.20 (0.66–2.22)
Age		1.00 (1.00–1.00)
PRISM III		1.01 (0.99–1.04)
Immunocompromised state		3.26 (2.03–5.38)
Multi-comorbidity		2.08 (1.17–3.72)
Delta HRVi Measurement	0.65 (0.45–0.93)	0.55 (0.36–0.82)
Age		1.00 (0.99–1.00)
PRISM III		1.01 (0.99–1.04)
Immunocompromised state		3.11 (1.94–5.10)
Multi-comorbidity		2.38 (1.32–4.31)

### Secondary Outcome

The total number of re-hospitalizations in the year after the index admission were analyzed as count data. The median last HRVi and delta HRVi measurements were significantly associated with the total number of re-hospitalizations after adjusting for age, PRISM III, immunocompromised state, and multi-comorbidity using Poisson regression, with higher HRVi measurements associated with a lower adjusted incidence rate ratio of a re-hospitalization. The lowest HRVi measurement was also associated with the number of re-hospitalizations with a higher value associated with increased likelihood of more re-hospitalizations (Table 4).

**Table 4:** Unadjusted and adjusted incidence rate ratios (IRR) for the number of re-hospitalizations within 1 year after septic shock admission based on age-normalized HRVi.

**Predictor**	**Unadjusted**	**Adjusted**
	**IRR (95% CI)**	**IRR (95% CI)**
Last HRVi Measurement	0.80 (0.76–0.84)	0.80 (0.77–0.84)
Age		1.00 (1.00–1.00)
PRISM III		1.02 (1.02–1.02)
Immunocompromised state		2.18 (2.09–2.28)
Multi-comorbidity		2.25 (2.06–2.48)
Lowest HRVi Measurement	1.35 (1.29–1.42)	1.54 (1.45–1.64)
Age		1.00 (1.00–1.00)
PRISM III		1.03 (1.02–1.03)
Immunocompromised state		2.01 (1.92–2.10)
Multi-comorbidity		2.28 (2.08–2.50)
Delta HRVi Measurement	0.66 (0.63–0.69)	0.63 (0.60–0.66)
Age		1.00 (1.00–1.00)
PRISM III		1.02 (1.02–1.03)
Immunocompromised state		2.03 (1.94–2.12)
Multi-comorbidity		2.40 (2.19–2.64)

### Sensitivity Analysis

In a sensitivity analysis of patients re-hospitalized within 30 or 90 days of the index admission, the delta HRVi remained significantly associated with re-hospitalization, with a higher value associated with a lower likelihood of readmission (*p* < 0.001). The median last HRVi was not significantly associated with re-hospitalization at 30 days, while the median lowest HRVi had positive, statistically significant association with re-hospitalization at 30 and 90 days (Supplementary Tables 2, 3).

## Discussion

In this study we demonstrate that autonomic nervous system dysfunction (ANSD) measured using age-normalized integer heart rate variability (HRVi) is independently associated with re-hospitalization in pediatric septic shock survivors. Specifically, the delta HRVi between the day with the lowest HRVi and the last day of PICU during the index hospitalization was significantly associated with re-hospitalization within 1 year, as well as within 30 and 90 days in sensitivity analysis, with a smaller recovery in the HRVi trajectory associated with higher likelihood of re-hospitalization. This association remained significant after controlling for several potential confounders, including age, PRISM III score, immunocompromised state, and multi-comorbidity. The absolute HRVi mean values on the lowest and last day of PICU stay were inconsistently associated with the outcome across the primary endpoint of readmissions at 1 year and the sensitivity analyses at 30 and 90 days. This suggests that the relative trajectory of recovery of the HRVi at the patient level was more predictive of re-hospitalization than the absolute values at a population level. Furthermore, although commonly considered confounders of poor outcomes, the severity of illness on admission based on PRISM III, patient age, duration of mechanical ventilation and vasoactive-inotrope days did not differ between patients requiring re-hospitalization and those who did not. This lack of association between common physiologic and clinical variables during the index admission and our outcome of interest (re-hospitalizations) makes the independent association between the recovery trajectory of HRVi during PICU admission and re-hospitalization all the more notable.

Readmissions after sepsis are common in children. Our rates of re-hospitalization of 66% and hospital readmission of 58% within a year after septic shock are consistent with prior studies. In a state-wide analysis of pediatric severe sepsis survivors, 47% of children were readmitted within 3 months and 85% of these readmissions were considered urgent or emergent (21). In another multicenter observational cohort study, almost 20% of severe sepsis survivors were readmitted within 30 days, and 30% within 90 days (22). Among children with severe sepsis, those with oncologic, hematologic, immunologic, and neurologic comorbidities were at higher risk of readmission (21). Additionally, children with age <1 year and with cardiovascular, bloodstream and genitourinary infections were at higher risk of readmission (21). In our population, 57% of pediatric septic shock survivors were re-hospitalized with recurrent infection, respiratory illness, or hematologic/oncologic diagnoses.

Health-related quality of life (HRQL) among pediatric sepsis survivors frequently declines, with 17–35% of sepsis survivors demonstrating more disability at discharge when compared to admission (2, 6). This decline includes functional and neuropsychological disability (23), and in some patients can persist for years (24). A retrospective national cohort study of over 9,000 pediatric patients examined potentially preventable readmissions following hospitalization for severe sepsis and other acute care conditions. They found that preventable readmissions were statistically higher following a severe sepsis admission when compared to other acute care admissions. Additionally, that study found 30-day readmissions were lower in patients with no comorbidities (5.9%) when compared to patients with co-morbidities (26%) (22).

While pre-existing comorbidities have been shown to be a driver of readmission following sepsis, our data support that other measures, such as HRVi, are worth considering. In our study, delta HRVi remained significantly associated with re-hospitalization even after controlling for multi-comorbidity and across sensitivity analyses. HRVi is a continuous, non-invasive physiologic marker of ANS function and can be measured in virtually all critically ill patients with high-frequency data collected from the bedside monitor. HRV has been shown to be predictive of in-hospital mortality in adults with sepsis (25) and in critically ill children (11). Our data now suggest that it can also be used to predict re-hospitalization in pediatric septic shock survivors. Real-time measurement of HRVi throughout the admission, and at discharge, may improve identification of patients who are at highest risk of re-hospitalization and facilitate the implementation of strategies to minimize this risk. Furthermore, because HRVi requires only a lightweight implementation, it is conceivable that this physiologic marker could be monitored in septic shock survivors after they are discharged from the hospital using wearable sensors like smart watches.

Our study has several strengths and limitations. First, this was a single center study and therefore may have limited generalizability. However, we present data on a relatively large cohort of septic shock survivors with available high-frequency physiologic bedside monitor data. Second, our definition of re-hospitalization only included patients who were re-hospitalized to our institution. It is possible that some patients were readmitted to other institutions. Third, we calculated the HRVi using high-frequency integer heart rate data, which is a lightweight proxy of HRV. While this method may be more generalizable than the derivation of HRV from R-R intervals in the electrocardiogram waveform, which is more computationally intense, HRVi may not fully capture the physiologic complexity of the ANS. Of note, we were unable to examine medications or specific conditions that may directly affect the autonomic nervous system. Finally, there may have been bedside monitor data losses during the study period, most commonly due to server downtimes without back-ups. We do not believe these data losses resulted in systematic bias in the study population since they occurred at random.

In conclusion, our data demonstrate a significant association between HRVi recovery and re-hospitalization among survivors of pediatric septic shock. This continuous, non-invasive physiologic marker of ANS function may be useful in the derivation of prediction models to stratify and mitigate the risk of re-hospitalization in survivors of pediatric septic shock.

## Data Availability Statement

The raw data supporting the conclusions of this article will be made available by the authors without undue reservation.

## Ethics Statement

The studies involving human participants were reviewed and approved by Ann & Robert H. Lurie Children's Hospital of Chicago Institutional Review Board. Written informed consent from the participants' legal guardian/next of kin was not required to participate in this study in accordance with the national legislation and the institutional requirements.

## Author Contributions

CB and LS-P designed the study and performed the data analysis. All authors made substantial contributions to drafting and final approval of the manuscript and agree to be accountable for the content of the work.

## Conflict of Interest

The authors declare that the research was conducted in the absence of any commercial or financial relationships that could be construed as a potential conflict of interest.

## Publisher's Note

All claims expressed in this article are solely those of the authors and do not necessarily represent those of their affiliated organizations, or those of the publisher, the editors and the reviewers. Any product that may be evaluated in this article, or claim that may be made by its manufacturer, is not guaranteed or endorsed by the publisher.

## Abbreviations

ANS: autonomic nervous system
ANSD: autonomic nervous system dysfunction
HRQL: health-related quality of life
HRV: heart rate variability
HRVi: integer heart rate variability
PRISM: Pediatric Risk of Mortality.
