# Supplementary material for: Autonomic Nervous System Dysfunction Is Associated With Re-hospitalization in Pediatric Septic Shock Survivors
Source: Front Pediatr. 2022 Jan 4;9:745844. doi: 10.3389/fped.2021.745844 (PMC8764397; doi:10.3389/fped.2021.745844)
Supplement: Supplementary file 1 [file Data_Sheet_1.docx]

Autonomic Nervous System Dysfunction is Associated with Re-Hospitalization in Pediatric Septic Shock Survivors

Colleen M. Badke, Lindsey Swigart, Michael S. Carroll, Debra E. Weese-Mayer, L. Nelson Sanchez-Pinto

**SUPPLEMENTAL DIGITAL CONTENT**

**TABLE OF CONTENTS**

**Digital File** **Page**

Supplemental Table 1 3

Supplemental Table 2 4

Supplemental Table 3 5

Supplemental Table 4 6

**Supplemental Table 1. Reason for re-hospitalization**

| **Diagnosis** | **1 year**  **Re-hospitalization**  **(n=306)** | **90-Day**  **Re-hospitalization**  **(n=207)** | **30-Day**  **Re-hospitalization**  **(n=85)** |
| --- | --- | --- | --- |
| Respiratory | 64 (21%) | 40 (19%) | 9 (11%) |
| Infection/Sepsis | 56 (18%) | 36 (17%) | 15 (18%) |
| Hematologic/Oncologic | 55 (18%) | 50 (24%) | 30 (35%) |
| Gastrointestinal | 47 (15%) | 26 (13%) | 12 (14%) |
| Neurologic | 26 (8%) | 14 (7%) | 7 (8%) |
| Metabolic | 13 (4%) | 10 (5%) | 3 (4%) |
| Other/Unknown | 45 (15%) | 31(15%) | 9 (11%) |

**Supplemental Table 2.** **Sensitivity analysis of** **HRVi measurements for septic shock survivors: 30 and 90-day re-hospitalization**

|  | **Not Re-hospitalized** | **Re-hospitalized within 30 days** | **P-value*** |
| --- | --- | --- | --- |
| **Last HRVi, median (IQR)** | -0.299 (-0.609, 0.058) | -0.305 (-0.495, -0.065) | 0.818 |
| **Lowest HRVi, median (IQR)** | -1.019 (-1.267, -0.624) | -0.607 (-0.848, -0.342) | <0.001 |
| **Delta HRVi, median (IQR)** | 0.570 (0.214, 1.020) | 0.213 (0.083, 0.465) | <0.001 |
|  | **Not Re-hospitalized** | **Re-hospitalized within 90 days** | **P-value*** |
| **Last HRVi, median (IQR)** | -0.248 (-0.570, 0.070) | -0.367 (-0.607, -0.054) | 0.02 |
| **Lowest HRVi, median (IQR)** | -1.012 (-1.271, -0.630) | -0.848 (-1.167, -0.470) | 0.001 |
| **Delta HRVi, median (IQR)** | 0.596 (0.218, 1.076) | 0.383 (0.116, 0.739) | <0.001 |

*Kruskal-Wallis test. P<0.05 considered significant.

**Supplemental Table 3. Unadjusted and adjusted odds ratios (OR) for re-hospitalizations within 30 days after septic shock admission based on age-normalized HRVi (n=85)**

| **Predictor** | **Unadjusted OR (95% CI)** | **Adjusted OR (95% CI)** |
| --- | --- | --- |
| Last HRVi Measurement | 1.00 (0.60-1.63) | 0.88 (0.49-1.52) |
| Age |  | 1.00 (1.00-1.01) |
| PRISM III |  | 0.94 (0.91-0.97) |
| Immunocompromised state |  | 5.22 (3.11-8.95) |
| Multi-comorbidity |  | 0.50 (0.25-1.01) |
| Lowest HRVi Measurement | 7.07 (3.89-13.25) | 4.44 (2.17-9.26) |
| Age |  | 1.00 (1.00-1.00) |
| PRISM III |  | 0.97 (0.93-1.00) |
| Immunocompromised state |  | 4.11 (2.41-7.14) |
| Multi-comorbidity |  | 0.55 (0.28-1.13) |
| Delta HRVi Measurement | 0.18 (0.09-0.34) | 0.29 (0.14-0.57) |
| Age |  | 1.00 (1.00-1.00) |
| PRISM III |  | 0.95 (0.92-0.99) |
| Immunocompromised state |  | 4.50 (2.66-7.78) |
| Multi-comorbidity |  | 0.63 (0.31-1.28) |

**Supplemental Table 4. Unadjusted and adjusted odds ratios (OR) for re-hospitalizations within 90 days after septic shock admission based on age-normalized HRVi (n=207)**

| **Predictor** | **Unadjusted OR (95% CI)** | **Adjusted OR (95% CI)** |
| --- | --- | --- |
| Last HRVi Measurement | 0.66 (0.44-0.97) | 0.59 (0.39-0.89) |
| Age |  | 1.00 (1.00-1.00) |
| PRISM III |  | 0.98 (0.96-1.00) |
| Immunocompromised state |  | 2.64 (1.77-3.98) |
| Multi-comorbidity |  | 1.51 (0.85-2.75) |
| Lowest HRVi Measurement | 2.05 (1.32-3.22) | 2.18 (1.24-3.87) |
| Age |  | 1.00 (1.00-1.00) |
| PRISM III |  | 1.00 (0.97-1.02) |
| Immunocompromised state |  | 2.26 (1.50-3.43) |
| Multi-comorbidity |  | 1.52 (0.85-2.76) |
| Delta HRVi Measurement | 0.43 (0.29-0.63) | 0.40 (0.26-0.61) |
| Age |  | 1.00 (1.00-1.00) |
| PRISM III |  | 0.99 (0.97-1.02) |
| Immunocompromised state |  | 2.31 (1.54-3.49) |
| Multi-comorbidity |  | 1.74 (0.96-3.20) |
